# Microbiota differences of skin and pharyngeal microbiota between patients with plaque and guttate psoriasis in China

**DOI:** 10.3389/fmicb.2022.937666

**Published:** 2022-08-10

**Authors:** Jingjin Hu, Wenhua Lu, Xingyu Li, Jing Yang, Minjia Tan, Kun Hu, Qiaolin Wang, Sichun Deng, Yijie Liu, Junchen Chen, Wu Zhu, Yehong Kuang

**Affiliations:** ^1^The Department of Dermatology, Xiangya Hospital, Central South University, Changsha, China; ^2^National Clinical Research Center for Geriatric Disorders, Xiangya Hospital, Changsha, China; ^3^Hunan Engineering Research Center of Skin Health and Disease, Xiangya Hospital, Changsha, China; ^4^Hunan Key Laboratory of Skin Cancer and Psoriasis, Xiangya Hospital, Changsha, China; ^5^The Dermatology of Taiyuan Central Hospital of Shanxi Medical University, Jinzhong, China

**Keywords:** skin, pharynx, microbiota, psoriasis area and severity index, plaque psoriasis, guttate psoriasis

## Abstract

Psoriasis can be provoked or exacerbated by environmental exposures such as certain microbiomes. The distinction between plaque psoriasis (PP) and guttate psoriasis (GP) in the skin or pharyngeal microbiota is not yet clear. High-throughput sequencing using Illumina MiSeq was used in this study to characterize skin and pharyngeal microbial composition in patients with PP [large PP (LPP, *n* = 62), small PP (SPP, *n* = 41)] and GP (*n* = 14). The alpha- and beta-diversity of skin microbiota LPP was similar to that of the SPP group, but different from the GP group. There were no differences in pharyngeal microbiota among the groups. According to linear discriminant analysis effect size (LEfSe) analysis, *Staphylococcus*, *Stenotrophomonas*, *Enhydrobacter*, *Brevundimonas*, and *Allorhizobium*–*Neorhizobium*–*Pararhizobium*–*Rhizobium* were the dominant genera of skin microbiota in PP. Diversity of skin microbiota correlated with Psoriasis Area and Severity Index (PASI). Moderate-to-severe psoriasis and mild psoriasis have different microbiota compositions. The skin microbiota may be related to the pharyngeal microbiota. Furthermore, two microbiota-based models could distinguish psoriasis subtypes with area under the receiver-operating characteristic curve (AUC-ROC) of 0.935 and 0.836, respectively. In conclusion, the skin microbiota in patients with LPP is similar to that in patients with SPP, but displays variations compared to that of GP, no differences are noted between subtypes in pharyngeal microbiota. Skin microbiota diversity correlated with PASI.

## Introduction

Psoriasis is a chronic inflammatory skin disease contributed by complex genetic and environmental factors, with at least 100 million individuals affected worldwide (Parisi et al., 2013; World Health Organization, 2016; Griffiths et al., 2021). Plaque psoriasis (PP) is the most common variant subtype of this disease (Griffiths et al., 2021), classified into two types based on the size of the lesions and the thickness of the plaques: small PP (SPP) and large PP (LPP; Griffiths et al., 2007). Psoriasis can also manifest as guttate psoriasis (GP), which often occurs in young children and adults. Of the environmental factors believed to induce or exacerbate psoriasis, microbes are critically involved in psoriasis (Waldman et al., 2001; Langan et al., 2018; Sbidian et al., 2019).

Skin microbiota consists of resident and transient flora and varies dynamically across space and time (Costello et al., 2009). Its interaction with host coordinates skin homeostasis (Sanford and Gallo, 2013; Chen et al., 2018). The dysbiosis of skin microbiota has been proposed as a possible cause of chronic cutaneous inflammatory diseases, such as atopic dermatitis and psoriasis (Fyhrquist et al., 2019). In one large cohort study, for example, the diversity and richness of the skin’s microbiota were significantly decreased in psoriatic lesions compared to that of psoriatic skin that was unaffected and in controls (Alekseyenko et al., 2013). Nowadays, it is demonstrated that skin microbiota dysbiosis in psoriasis results in disordered keratinocyte signaling and predisposition to IL-17, which drives a pathogenic IL-17 loop (Dainichi et al., 2018) or may exacerbate the disease (Chen et al., 2020).

Pharynx (referred as throat in Human Microbiome Project) is constantly exposed to large quantities of inhaled and ingested microbes and is also an important site of pathogen colonization (Lemon et al., 2010). The colonized microorganisms interact and coexist with the local mucosal immune system of the respiratory tract, maintaining the immune stability and balance of the respiratory system (Guo et al., 2021). Previous research indicates that psoriasis (especially GP) is commonly triggered by an acute *Streptococcus pyogenes* (*S. pyogenes*) infection of the throat or tonsils (Telfer et al., 1992; Fry et al., 2013). Furthermore, a study suggested that in patients with PP, IgA plasma levels against *S. pyogenes* were correlated with cutaneous lymphocyte-associated antigen + T-cell-dependent IL-17F response *in vitro* (De JESús-Gil et al., 2020). However, significant alpha- and beta-diversity differences in pharyngeal microbiota were not detected between patients with psoriasis and healthy controls in another recent study (Assarsson et al., 2020).

Although the skin microbiota has been frequently studied in psoriasis, the pharyngeal microbiota in psoriasis has received far less attention (Alekseyenko et al., 2013; Chang et al., 2018; Quan et al., 2020). Unlike the known relationship between the microbiome and dermatologic diseases, such as *Staphylococcus aureus* (*S. aureus*) with atopic dermatitis (Boguniewicz and Leung, 2011), *Cutibacterium acnes* with acne vulgaris (Williams et al., 2012), and *Malassezia furfur* with seborrheic dermatitis (Hay, 2011), there is little consensus yet regarding specific bacterial taxa defining psoriasis. Furthermore, there is a paucity of research on differences in the skin and pharyngeal microbiota among different subtypes of psoriasis, as well as the relationship between differential microbiota and psoriasis severity. This cross-sectional study analyzed the skin and pharyngeal microbiota of patients with LPP, SPP, and GP by high-throughput sequencing of the 16S rRNA gene, aiming to provide a theoretical basis for differential diagnosis and pathogenesis of psoriasis subtype.

## Materials and methods

### Study participants

Patients with psoriasis were prospectively recruited from the Dermatology Department of Xiangya Hospital of Central South University from January 2018 to December 2019. This study was approved by the Ethics Committee of the Xiangya Hospital of Central South University (2018121106). All the participants in this trial have signed the informed consent, and their demographic data, clinical presentation, and medical history were then recorded. The diagnosis of psoriasis was confirmed using clinical or/and histopathological criteria. In accordance with the size of lesions and thickness of plaques, the patients were grouped in LPP (at least one lesion with a long axis of ≥ 5 cm), SPP (1–5 cm lesions), and GP (<1 cm lesions). Patients included current active psoriasis of the skin: with no psoriatic arthritis, erythroderma, or pustular psoriasis; willingness to avoid facial washing; and application of topical agents to the face for 24 h before skin sampling. Exclusion criteria were as follows: without lesion area on tibialis anterior of lower limbs; recent (<4 weeks prior) use of any systemic psoriasis therapy (e.g., antibiotics, steroids, retinoic, methotrexate, cyclosporine, and biologic drugs); certain topical psoriasis treatment or phototherapy (<2 weeks prior), bath therapy (within 24 h); with comorbid skin and soft tissue infection or other skin diseases; and with comorbid autoimmune diseases, diabetes mellitus, and coagulopathy. A total of 117 psoriasis subjects (62 with LPP, 41 with SPP, and 14 with GP) who met study criteria were offered enrollment (Supplementary Figure 1).

### Clinical characteristics

The following characteristics were assessed as possible microbiome shaping factors: age, gender, marital status, educational level, smoking status, smoking status, BMI categories, waist-to-hip ratio (WHR), and psoriasis characterizations [age at onset, duration of disease, family history, body surface area (BSA), psoriasis area and severity index (PASI; Bożek and Reich, 2017), and the dermatology life quality index (DLQI; Lewis and Finlay, 2004)]. In childhood, BMI categories were defined using the Centers for Disease Control and Prevention (CDC) 2000 growth charts (Kuczmarski et al., 2002) as underweight (<5th percentile), normal weight (5th–85th percentile), and overweight/obese (≥ 85th percentile). In the adult, BMI categories were defined based on the World Health Organization classification (World Health Organization, 2000) as underweight (<18.5 kg/m^2^), normal weight (18.5–24.9 kg/m^2^), and overweight/obese (≥25 kg/m^2^).

### Sample collection

Samples were collected from the pharynx and the representative lesion skin from the tibialis anterior of either lower limb in patients with psoriasis with no prior cleaning. The skin target area was divided into four quadrants. The skin in each quadrant was repeatedly swabbed 5 times with a sterile cotton swab, then rotated the swab by 90, 180, and 270° to repeat the above steps. For the small area of skin lesion (GP), we swabbed multiple skin lesions with a sterile cotton swab. To collect the pharyngeal samples, an oropharyngeal swab was taken by swabbing and twirling the rear pharyngeal walls 2–3 times. Then, the cotton head was cut off at the indicated break line, placed in the preservation tube immediately, transported on ice, and then transferred to the − 80°C refrigerator within 2 h until deoxyribonucleic acid (DNA) extraction.

### Deoxyribonucleic acid extraction and 16S rRNA gene sequencing

Total genomic DNA was extracted using Protease K splitting (Merck, Germany) and Phenolic chloroform extraction (Generay Biotech, Shanghai, China). We PCR-amplified in duplicate the V3–V4 variable region of the bacterial 16S rRNA genes using specific primers 357 F (5′-ACTCCTACGGRAGGCAGCAG-3′) and 806 R (5′-GGACTACHVGGGTWTCTAAT-3′). The first step of PCR was performed by the following program: initial denaturation at 94°C for 2 min, followed by 25 cycles of denaturation at 94°C for 30 s, annealing at 56°C for 30 s and elongation at 72°C for 30 s, and finally, extension at 72°C for 5 min. The second step PCR with dual 8-base barcodes was used for multiplexing. Eight-cycle PCRs were used to incorporate two unique barcodes to either end of the 16S amplicons. Cycling conditions consisted of one cycle of 94°C for 3 min, followed by 8 cycles of 94°C for 30 s, 56°C for 30 s, and 72°C for 30 s, followed by a final extension cycle of 72°C for 5 min. Prior to library pooling, the barcoded PCR products were purified using a DNA Gel Extraction Kit (Axygen Biosciences, Union City, CA, United States) and quantified using FTC-3000TM Real-Time PCR (Funglyn, Shanghai, China) according to the manufacturer’s protocol. The PCR products from different samples were indexed and mixed at equimolar ratios for sequencing on the Illumina platform at TinyGene Bio-Tech (Shanghai, China) Co., Ltd.

### Bioinformatic analysis

Purified amplicons were paired-end sequenced (2 × 300) on an Illumina MiSeq platform (Illumina, San Diego, United States) according to the standard protocols by TinyGene Bio-Tech (Shanghai, China) Co., Ltd. (Fadrosh et al., 2014). Due to either skin or pharyngeal samples failed sequencing, the paired sequencing samples from 8/125 patients were excluded from subsequent analysis. After that, the paired-end reads were assigned to samples based on their unique barcode and truncated by cutting off the barcode and primer sequence. Paired-end reads were merged using FLASH program (version 1.2.11; Magoè and Salzberg, 2011). To obtain the high-quality clean tags, raw tags with low quality (quality value ≤ 19, homopolymers ≥ 3 bases, and sequence length ≤ 75% of the tags) were filtered by QIIME (version 1.9.1; Caporaso et al., 2010). The clean tags were compared with the Silva database using UCHIME (Edgar et al., 2011) to detect and remove chimera sequences. Then the effective tags were obtained.

Operational taxonomic units (OTUs) clustering was performed using Uparse (version 7.0.1001^1^; Edgar, 2013) at a minimum of 97% confidence threshold. After filtering, the resulting dataset consisted of 2,178 OTUs with an average of 34,940 reads per sample (Supplementary Excel 1). Phylogenetic classification was performed using the Bayesian classifier algorithm against the 16S rRNA database, Silva SSU128 (Quast et al., 2013), with a boot-strap cutoff value of 60%, and 1,567 (71.95%) OTUs annotated to the genus level.

The obtained OTU data were analyzed in R software (version 3.4.1). The richness and α-diversity of microbial composition were assessed through Observed species, chao1, Shannon index, and phylogenetic distance (PD) whole tree, calculated by the QIIME software (version 1.9.1). Analysis of variance (ANOVA) was performed to evaluate α-diversity among the different groups. β-diversity analysis was used to compare the composition of the skin and pharyngeal microbiota in each group, and principal coordinate analysis (PCoA) and permutational multivariate ANOVA (PERMANOVA) test were calculated by weighted and unweighted UniFrac distance method (Lozupone and Knight, 2005). The linear discriminant analysis (LDA) effect size (LEfSe) method was used to characterize the taxa with statistical significance and biological relevance (Segata et al., 2011). For the LEfSe analysis, the Kruskal–Wallis test (α-value of 0.05) and LDA score of > 4 were used as thresholds. Pairwise comparisons were analyzed with the Mann–Whitney *U*-test. Using PICRUSt2 (Douglas et al., 2020), we predicted the functional difference in relative abundance on Kyoto Encyclopedia of Genes and Genomes (KEGG) level 3 pathways that were present in at least 15% of samples, and with a minimum mean relative abundance of 0.01%. The Wilcoxon rank-sum test was employed and then followed by Benjamini–Hochberg false discovery rate (FDR) correction (Yoav and Yosef, 1995). The Spearman correlation analysis was performed to demonstrate the relationship between skin microbiota and clinical characteristics.

### Statistical analysis

All results were presented as mean with standard deviation (mean ± SD), numbers with percentages, or medians with interquartile range [medians (IQR)]. Comparisons were conducted using the Student’s *t*-test or Mann–Whitney *U*-test. Multiple comparisons were performed by one-way ANOVA (consistent with normal distribution and variance) followed a Bonferroni multiple comparisons correction or non-parametric statistics (not conforming to normal distribution or variance). For correlation analysis, the Spearman rank test was performed. The Pearson (ρ) and Kendall (τ) rank correlation coefficients were used to investigate the associations between Shannon index and clinical characteristics, as appropriate. Receiver operating characteristic (ROC) analysis was used to evaluate the diagnostic accuracy of differential microbiota based on the LEfSe analysis and summarized as area under the ROC (AUC) with corresponding 95% confidence intervals. All statistical analyses and graphing were performed using IBM SPSS (Version 26.0, NY: IBM Corp, United States). A *p*-value < 0.05 was considered a significant difference.

### Data availability statement

Amplicon sequencing clean data have been deposited at the NCBI Sequence Read Archive (SRA)^2^ under accession number PRJNA835606.

## Results

### Demographics and clinical characteristics

In total, 117 eligible psoriasis subjects (62 with LPP, 41 with SPP, and 14 with GP) were included in this study according to the recruitment process. It was reported that GP accounts for 1–2% of the total cases of psoriasis, which was consistent with the included proportion (Ogawa et al., 2018). Detailed demographics and clinical characteristics are described in Table 1. As shown, positive values indicated that patients with LPP were older (*p* < 0.001), more men (*p* = 0.025), more married or divorced (*p* < 0.001), and had a higher BMI (*p* = 0.009), a higher WHR (*p* = 0.008), and later age at onset (*p* = 0.008) than that of the patients with SPP and GP. To reduce the negative impact of missing data, we eliminated two missing data on PASI, BAS, and DLQI in the GP group and compared the differences of the disease activity scores among three groups. As expected, subjects with LPP had higher PASI, BSA, and DLQI scores compared to the SPP and GP groups, but no difference between SPP and GP groups. There was no significant difference in the other characteristics between the three groups.

**TABLE 1:** Demographics and clinical characteristics of the participating subjects.

Characteristics	Total PsO (*N* = 117)	LPP (*N* = 62)	SPP (*N* = 41)	GP (*N* = 14)	*P*-value (LPP vs. SPP vs. GP)
**Demographics**					
Age, y, mean (SD)	39.5 (13.6)	43.8 (12.0)^a^	37.7 (13.5)^b^	26.0 (11.2)^c^	0.000*** a > b > c
Gender					0.025*
Male	66 (56.4%)	42 (67.7)	19 (46.3)	5 (35.7%)	
Female	51 (43.6%)	20 (32.3%)	22 (53.7%)	9 (64.3%)	
Marital status					0.000***
Unmarried	28 (23.9%)	9 (14.5%)	10 (24.4%)	9 (64.3%)	
Married/divorced	89 (76.1%)	53 (85.5%)	31 (75.6%)	5 (35.7%)	
Educational level					0.146
≤Elementary school	20 (17.1%)	10 (16.1%)	8 (19.5%)	2 (14.3%)	
High school	64 (54.7%)	38 (61.3%)	22 (53.7%)	4 (28.6%)	
≥College	33 (28.2%)	14 (22.6%)	11 (26.8%)	8 (57.1%)	
Smoking status					0.463
Never smoker	63 (53.8%)	30 (48.4%)	24 (58.5%)	9 (64.3%)	
Former smoker	12 (10.3%)	9 (14.5%)	2 (4.9%)	1 (7.1%)	
Current smoker	42 (35.9%)	23 (37.1%)	15 (36.6%)	4 (28.6%)	
Drinking status					0.515
No	82 (70.1%)	44 (71.0%)	30 (73.2%)	8 (57.1%)	
Yes	35 (29.9%)	18 (29.0%)	11 (26.8%)	6 (42.9%)	
BMI categories, n (%)					0.009**
Underweight	9 (7.7%)	1 (1.6%)	4 (9.8%)	4 (28.6%)	
Normal weight	79 (67.5%)	41 (66.1%)	29 (70.7%)	9 (64.3%)	
Overweight/Obese	29 (24.8%)	20 (32.3%)	8 (19.5%)	1 (7.1%)	
WHR, mean (SD)^‡^	0.90 (0.07)	0.91 (0.68)^a^	0.89 (0.76)^b^	0.85 (0.05)^c^	0.008** a > c
**Psoriasis characterization**					
Age at onset, y, mean (SD)	29 (12.7)	33 (12.6)^a^	27 (12.5)^b^	20 (8.4)^c^	0.002^**^ a > c
Duration of psoriasis, y, mean (SD)	9.2 (8.2)	10.3 (8.0)	9.1 (8.9)	5.5 (6.8)	0.147
Family history of psoriasis					0.229
No	97 (82.9%)	48 (77.4%)	37 (90.2%)	12 (85.7%)	
Yes	20 (17.1%)	14 (22.6%)	4 (9.8%)	2 (14.3%)	
PASI, mean (SD) ^†^	12.1 (9.6)	16.3 (10.3)^a^	8.0 (6.8)^b^	5.4 (2.7)^c^	0.000*** a > b, c
BSA, mean (SD) ^†^	16.9 (16.9)	22.7 (18.5)^a^	11.2 (13.5)^b^	7.5 (5.2)^c^	0.000*** a > b, c
DLQI, mean (SD) ^†^	7 (4)	8 (4)^a^	5 (4)^b^	6 (5)^c^	0.003** a > b, c

^a,b,c^ Results of the Bonferroni pairwise comparison after one-way ANOVA are reported by the different superscript letters. Except where indicated otherwise, values are the number (%) of subjects. ^‡^The mean imputation was used to replace two missing values with the mean value of three neighbor waist-to-hip ratio as per the BMI in ascending order. ^†^Missing data on PASI, BAS, and DLQI in the GP group (*n* = 2). BMI, body mass index; WHR, waist-to-hip ratio; PASI, psoriasis area and severity index; BSA, body surface area; DLQI, dermatology life quality index; GP, guttate psoriasis; LPP, large plaque psoriasis; SPP, small plaque psoriasis. *p < 0.05; **p < 0.01; ***p < 0.001; Bold font, significant difference.

### Microbiota diversity was different between psoriasis subtypes for the skin, but not for the pharynx

#### Sequence analysis and quality filtering

To detect whether the psoriasis subtypes were associated with a change in microbiota characteristics, we sequenced the hypervariable V3–V4 region of the lesion epidermal and throat swab samples utilizing an Illumina MiSeq System16S rRNA gene sequencing. Of these, 8,864,007 high-quality sequences were selected, yielding an average of 34,940 sequences per sample after filtering out low-quality reads (Supplementary Excel 1). After the OTUs clustering and species annotation, 2,177 (99.95%) effective OTUs were obtained with at least a 97% similarity level (Supplementary Excel 2). The species rarefaction curves demonstrated that all samples approached a saturating sequencing department as indicated by the curve plateaus (Supplementary Figure 2A). The species accumulation boxplot tended to flatten, indicating that the sampling effort was sufficient (Supplementary Figure 2B).

To better understand the shared richness in the skin or pharyngeal microbiota among the three groups, we constructed Venn diagrams illustrating the intersection between groups (Supplementary Figures 2C,D), which revealed the overlapped or unique OUTs of different groups. There were 677 (33.2%) overlapped OTUs of all groups in skin microbiota, and 450 (39.9%) in pharyngeal microbiota.

#### Microbiota taxonomic composition

The microbiota of the skin or pharynx of patients with psoriasis and healthy controls has already been demonstrated to differ significantly (Assarsson et al., 2020; Olejniczak-Staruch et al., 2021). In order to investigate the microbiota community structure of patients with PP and GP, we examined the relative abundances of LPP, SPP, and GP samples by stacked bar plots to determine the predominant composition among these groups (Supplementary Figures 3A,D). As is shown, a great predominance of phylum *Proteobacteria* (mean relative abundance = 60.2%) was found in the psoriasis skin microbiota, followed by *Firmicutes* (16.7%), *Actinobacteria* (9.8%), and *Bacteroidota* (8.4%) and the other phyla (relative abundance < 5%). The dominant bacterial genera in skin were *Pseudomonas* (mean relative abundance = 18.5%, *Proteobacteria*), *Halomonas* (7.4%, *Proteobacteria*), *Staphylococcus* (6.3%, *Firmicutes*), *Cutibacterium* (4.9%, *Actinobacteria*), *Streptococcus* (2.9%, *Firmicutes*), *Enhydrobacter* (2.7%, *Proteobacteria*), *Zoogloea* (2.5%, *Proteobacteria*), *Brevundimonas* (1.8%, *Proteobacteria*), etc. The top 100 relative abundance OTUs at the genus level was represented in the evolutionary tree (Supplementary Figure 4). In the pharynx, *Firmicutes* (31.2%), *Bacteroidota* (28.3%), and *Proteobacteria* (21.0%) were the three phyla dominating the pharyngeal microbial community. *Prevotella_7* (12.5%, *Bacteroidota*), *Streptococcus* (12.2%, *Firmicutes*), and *Neisseria* (11.8%, *Proteobacteria*) were the most abundant genera.

#### Alpha-diversity

**FIGURE 1:**
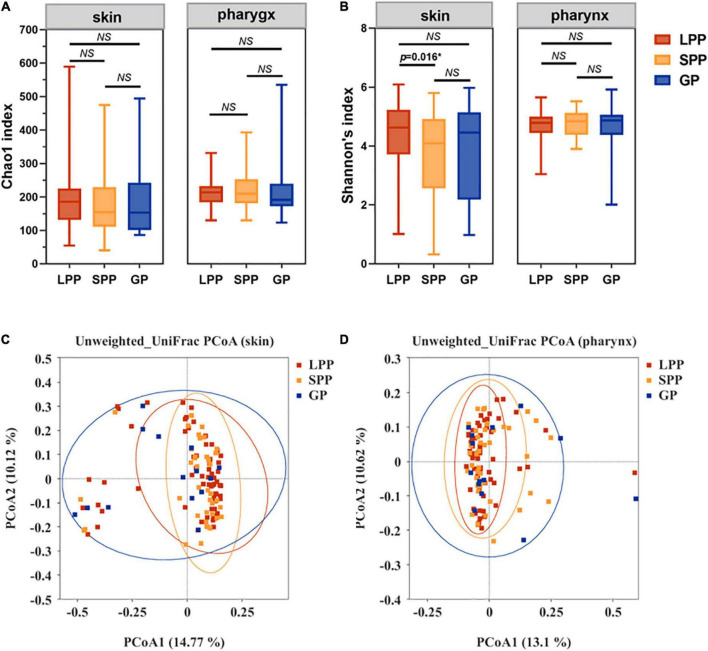
Comparison of the construction and diversity of the skin microbiota among the GP, SPP, and LPP subjects. **(A,B)** Alpha-diversity indices analysis based on indices of Chao1 **(A)** and Shannon **(B)** within each group of skin microbiota, respectively. Horizontal bars within boxes represent the median. The tops and bottoms of boxes represent the 75th and 25th quartiles separately. **(C,D)** Unweighted UniFrac PCoA **(C)** and Weighted UniFrac PCoA **(D)** plots of skin microbiota from the GP (green), SPP (blue), and LPP (red) groups. The points in different colors represented the skin microbial structure of the samples in each group. *p < 0.05. NS, no significance; PCoA, principal coordinate analysis.

To characterize the alpha-diversity of the bacterial communities among LPP, SPP, and GP groups, we calculated the Chao1 and Shannon index, respectively (Figures 1A,B and Supplementary Table 1). In the pharynx, there were no significant differences in alpha-diversity between groups. In the skin, no shifts in microbiota species richness were revealed by Chao1 index between groups (all *p*-values > 0.05). However, the diversity Shannon index of SPP groups tended to be lower than that of LPP groups (Wilcoxon test, *p* = 0.016), indicating that the skin microbiota in patients with SPP exhibited a lower diversity of species than patients with LPP.

#### Beta-diversity

To evaluate beta-diversity, namely the intergroup variability in skin or pharyngeal community structure, PCoA was performed based on the unweighted UniFrac distance method. Unweighted UniFrac Adonis and analysis of molecular variance (AMOVA) statistics were proceeded to further explain the community variations between groups (Table 2). The unweighted PCoA plot demonstrated that PCoA1 and PCoA2 accounted for 14.77 and 10.12% of the total variance, respectively (Figure 1C). The LPP group and the SPP group skin microbiota clustered together, whereas the plots belonging to the GP group were dispersed (Figure 1C). In the pharynx, the difference in beta-diversity between groups was similar to those of the skin microbiota, that is to say, there were differences in GP-LPP and GP-SPP intergroups, but no difference between SPP and LPP groups (Figure 1D).

**TABLE 2:** Adonis and Amova analyses based on unweighted UniFrac distance.

Sampling location	Versus_group	Adonis		AMOVA	
		*R*^2^ (R-value)	*P*-value	Fs	*P*-value
Skin					
	LPP vs SPP	0.011 (0.989)	0.298	1.100	0.275
	LPP vs GP	0.023 (0.977)	0.022*	1.770	0.022*
	SPP vs GP	0.033 (0.967)	0.010*	1.835	0.011*
Pharynx					
	LPP vs SPP	0.010 (0.990)	0.347	1.062	0.341
	LPP vs GP	0.025 (0.975)	0.020*	1.916	0.022*
	SPP vs GP	0.030 (0.970)	0.043*	1.64691	0.043*

GP, guttate psoriasis; LPP, large plaque psoriasis; SPP, small plaque psoriasis. *p < 0.05.

In summary, the microbial communities of the skin and pharynx of LPP and SPP groups were similar, but unlike those of the GP group. In accordance with the morphological classification of psoriasis, LPP and SPP belong to the PP subtype, but PP and GP are two psoriasis subtypes. Therefore, LPP and SPP groups were merged into the PP group to compare with the GP group in the further analysis. The intergroup comparisons revealed statistical differences between PP and GP groups in the skin microbiota assessed by the PERMANOVA test (unweighted *p* = 0.008, weighted *p* = 0.008). However, the intergroup difference in pharyngeal microbiota was significant only in unweighted UniFrac (unweighted *p* = 0.017, weighted *p* = 0.763), which suggested that the major differential microbiota between PP and GP groups was in the presence or absence and abundance of rare taxa.

### Microbiota dominance in patients with plaque psoriasis and guttate psoriasis

**FIGURE 2:**
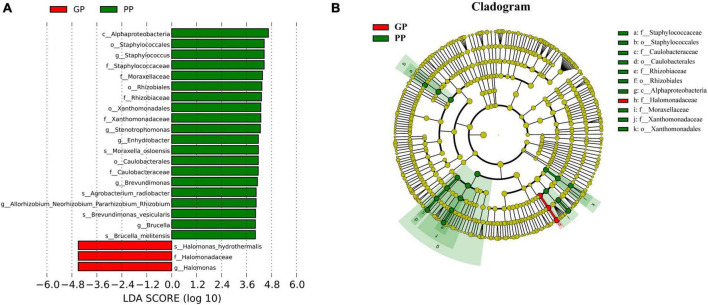
Composition and linear discriminant analysis of skin microbiota between PP and LPP groups. **(A)** Cladogram generated from the LEfSe analysis indicates the phylogenetic distribution of the microbiota of PP and GP groups from phylum to genus. **(B)** Histogram of LDA scores to identify differentially abundant bacteria between PP and GP. The threshold of linear discriminant analysis score was set as 4, and the larger the LDA score is, the greater differences among the three groups are.

To characterize taxonomic differences, we performed the Wilcoxon test on various levels of taxa between two groups (Table 3) and the histogram of LEfSe (LDA score > 4.0, *p* < 0.05; Figure 2). The LEfSe analysis showed that 21 bacterial clades at all taxonomic levels had differentially abundance between the PP and GP skin microbiota. No significant difference was observed in the top ten dominant phyla of the skin microbiota. Staphylococcaceae, Moraxellaceae, Rhizobiaceae, Xanthomonadaceae, and Caulobacteraceae were the dominant families in the PP group. The most dominant genera in the PP group were *Staphylococcus*, *Stenotrophomonas*, *Enhydrobacter*, *Brevundimonas*, and *Allorhizobium*–*-Neorhizobium*–*Pararhizobium*–*Rhizobium*. The family *Halomonadaceae*, its genus *Halomonas*, along with its species *Halomonas_hydrothermalis* were found to be characteristic microbiota in the GP group. Comparing within pharyngeal samples, the LEfSe analysis (LDA score > 4.0, *p* < 0.05, Supplementary Figure 5) revealed the order *Pseudomonadales* and family Halomonadaceae, its genus *Halomonas*, and its species *Halomonas_hydrothermalis* were specific microbiota in the GP group, whereas no predominant microbiota was found in the PP group. Overall, there were some changes between PP and GP in whether skin microbiota or pharyngeal microbiota. In contrast, the less differentially abundant microbiota of intergroup was detected in the pharyngeal samples. Interestingly, the genus *Halomonas* and its species *Halomonas_hydrothermalis* were the marked microbiota in the GP group in both skin and pharynx (Figure 2 and Supplementary Figure 5).

**TABLE 3:** Differences of relative abundance in the skin microbiota between GP and PP groups.

Taxa	GP group		PP group		*P*-value	*q*-value
	Mean (%)	SD (%)	Mean (%)	SD (%)		
*Family*						
Halomonadaceae	13.28	26.32	6.62	19.13	0.005	0.045
Staphylococcaceae	1.73	3.02	7.11	12.06	0.002	0.030
Rhizobiaceae	2.26	3.55	6.25	7.68	0.034	0.142
Moraxellaceae	0.59	0.50	5.33	8.47	0.000	0.007
Xanthomonadaceae	0.43	0.62	4.28	5.87	0.004	0.045
Bacteroidaceae	2.72	5.95	1.49	4.76	0.048	0.170
Caulobacteraceae	0.38	0.84	3.34	3.89	0.000	0.007
Weeksellaceae	0.16	0.16	1.89	3.84	0.017	0.093
Oxalobacteraceae	0.55	1.41	1.26	6.35	0.041	0.162
Rhodobacteraceae	0.13	0.16	1.55	4.09	0.006	0.045
*Genus*						
*Halomonas*	13.28	26.32	6.62	19.13	0.004	0.087
*Staphylococcus*	1.47	2.57	6.93	11.93	0.001	0.039
*Stenotrophomonas*	0.43	0.62	4.08	5.89	0.006	0.087
*Bacteroides*	2.72	5.95	1.49	4.76	0.048	0.288
*Enhydrobacter*	0.20	0.24	3.24	7.50	0.010	0.098
*Allorhizobium*–*Neorhizobium*–*Pararhizobium*–*Rhizobium*	0.40	1.23	2.61	3.85	0.005	0.087
*Brevundimonas*	0.14	0.31	2.84	3.95	0.001	0.039
*Aliihoeflea*	1.73	3.40	1.06	3.14	0.025	0.192
*Acinetobacter*	0.38	0.37	1.90	4.05	0.006	0.087
*Brucella*	0.07	0.19	2.19	3.55	0.008	0.087

Based on the Wilcoxon test, a non-parametric rank-sum test, we compared the dominant species at various levels of skin microbiota. GP, guttate psoriasis; PP, plaque psoriasis.

### The abundance of some taxa was positively correlated in the skin and pharynx

Later, we calculated the Spearman correlation coefficient to assess the correlations of bacterial taxa between the skin and pharyngeal habitats in patients with psoriasis. Positive correlations were observed between the skin and pharyngeal microbiota, including the phyla *Acidobacteriota* (ρ = 0.304, *p* = 0.001), *Bdellovibrionota* (ρ = 0.640, *p* < 0.001), and *Gemmatimonadota* (ρ = 1.000, *p* < 0.001) for all psoriasis individuals (PP + GP). The abundance of 53 genera was positively correlated between respective skin and pharyngeal samples (Table 4) in the PP or GP group. Among them, the genera *Prevotella_9*, *Bacteroides*, *Parabacteroides*, *Faecalibacterium*, *Eubacterium eligens* group, *Agathobacter*, and *Alistipes* were correlated in both the PP and GP groups. Interestingly, no negative correlations between the skin and pharyngeal microbiota were observed.

**TABLE 4:** The bacteria taxa whose abundance is correlated to skin and pharynx.

Patients with GP			Patients with PP		

Genus	rho	*p*	Genus	rho	*p*
*Dorea*	1.000	0	*Herbaspirillum*	0.741	<0.001
*Faecalibacterium* ^#^	0.998	<0.001	*Bdellovibrio*	0.665	<0.001
*Fusicatenibacter*	0.998	<0.001	*Xanthobacter*	0.663	<0.001
*Agathobacter* ^#^	0.997	<0.001	*Paracoccus*	0.632	<0.001
*Anaerostipes*	0.997	<0.001	*Sphingomonas*	0.592	<0.001
*Parabacteroides* ^#^	0.995	<0.001	*Halomonas*	0.575	<0.001
*Prevotella_9^#^*	0.995	<0.001	*Nesterenkonia*	0.562	<0.001
*Subdoligranulum*	0.995	<0.001	*Prevotella_9^#^*	0.560	<0.001
*Eubacterium hallii group*	0.992	<0.001	*Aliihoeflea*	0.536	<0.001
*Lachnospira*	0.992	<0.001	*Kingella*	0.479	<0.001
*Clostridium_sensu_stricto_1*	0.991	<0.001	*Abiotrophia*	0.462	<0.001
*Ruminococcus*	0.984	<0.001	*Cupriavidus*	0.421	<0.001
*Ruminococcus torques group*	0.982	<0.001	*Bacteroides* ^#^	0.391	<0.001
*Roseburia*	0.976	<0.001	*Lactobacillus*	0.375	<0.001
*Romboutsia*	0.974	<0.001	*Parabacteroides* ^#^	0.373	<0.001
*Sutterella*	0.973	<0.001	*Faecalibacterium* ^#^	0.370	<0.001
*Lachnospiraceae_NK4A136_group*	0.953	<0.001	*Alistipes* ^#^	0.352	<0.001
*Megamonas*	0.938	<0.001	*Parvimonas*	0.351	<0.001
*Peptoanaerobacter*	0.937	<0.001	*Candidatus_Saccharimonas*	0.312	0.001
*Curvibacter*	0.935	<0.001	*Eubacterium eligens group^#^*	0.241	0.014
*Bacteroides* ^#^	0.924	<0.001	*TM7x*	0.24	0.015
*Ruminococcus gnavus group*	0.909	<0.001	*Agathobacter* ^#^	0.237	0.016
*Lachnoclostridium*	0.908	<0.001	*Aggregatibacter*	0.224	0.023
*Blautia*	0.892	<0.001	*Anoxybacillus*	0.209	0.034
*Lentimicrobium*	0.873	<0.001	*Stomatobaculum*	0.200	0.043
*Mycoplasma*	0.832	<0.001			
*Klebsiella*	0.830	<0.001			
*Anaeroglobus*	0.810	<0.001			
*Ligilactobacillus*	0.790	0.001			
*Bifidobacterium*	0.764	0.001			
*Eubacterium eligens group^#^*	0.743	0.002			
*Eubacterium saphenum group*	0.741	0.002			
*Butyrivibrio*	0.734	0.003			
*Treponema*	0.717	0.004			
*Brevundimonas*	0.702	0.005			
*Lacibacter*	0.679	0.008			
*Zoogloea*	0.636	0.015			
*Fretibacterium*	0.586	0.028			
*Pelomonas*	0.585	0.028			
*Pseudopropionibacterium*	0.546	0.043			
*Alistipes* ^#^	0.540	0.046			

Spearman’s correlations and two-tailed *p*-value are displayed. Superscript “#” indicates the abundance of genera that were correlated in skin and pharynx in both the PP and GP groups. GP, guttate psoriasis; PP, plaque psoriasis.

### PICURSt2 function prediction of intergroup skin microbiota

To further predict the differential function of the microbial communities among the skin samples in two subtypes, 298 KEGG pathways were annotated by the KEGG pathway database, following which 231 with a mean relative abundance of at least 0.01%, and present in at least 15% of total samples, included in the follow-up analysis. Only 11 significantly enriched KEGG pathway categories were selected between PP and GP groups (Supplementary Table 2, *p* < 0.05 and FDR *q* < 0.15). It is important that seven pathways were upregulated in the PP group relative to the GP group, including “beta-Lactam resistance” (KEGG ID: 01501), “Oxidative phosphorylation” (KEGG ID: 00190), “Fatty acid biosynthesis” (KEGG ID: 00061), “Carotenoid biosynthesis” (KEGG ID: 00906), “Lipopolysaccharide biosynthesis” (KEGG ID: 00540), “Naphthalene degradation” (KEGG ID: 00626), and “Ethylbenzene degradation” (KEGG ID: 00642). “Ribosome biogenesis in eukaryotes” (KEGG ID: 03008), “Type II diabetes mellitus” (KEGG ID: 04930), “Methane metabolism” (KEGG ID: 00680), and “Lipoic acid metabolism” (KEGG ID: 00785) pathways were considered the enhanced pathways in the GP group.

### Association between skin microbiota community and psoriasis-related clinical parameters

**FIGURE 3:**
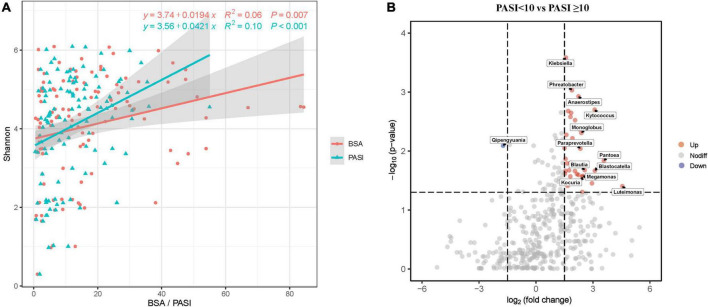
Skin microbial dysbiosis is associated with psoriasis PASI and BSA scores. **(A)** Scatter plot showing positive correlations between the Shannon index and PASI and BSA scores. Only statistically significant correlations are shown. Linear regression lines are shown in each scatter plot in red (BSA) and green (PASI), and shaded regions represent 95% CIs. **(B)** The volcano plots show the relative fold changes and whether they were significant. Assessed by the Wilcoxon rank-sum test, the *p*-values for each genus (or higher-level OTU) represent the statistical significance of abundance changes in the PASI ≥ 10 group compared with the PASI < 10 group. Red (increased) and green (decreased) bubbles indicate significant differential distribution (*p* < 0.05). PASI, psoriasis area and severity index; BSA, body surface area.

Due to skin microbiome compositional changes being associated with age, gender, BMI, and other factors (Giacomoni et al., 2009; Zhai et al., 2018; Michael et al., 2019), we explored the additional effects of demographic and clinical parameters on the skin microbiota. In univariate analyses based on the Pearson’s and Kendall’s tau correlations, the Shannon diversity index was positively associated with marital status (*p* = 0.013), BMI categories (*p* = 0.038), age at onset (*p* = 0.046), PASI (*p* < 0.001), and BSA (*p* = 0.007), but was not associated with age, gender, and the other variables (Supplementary Table 3). These variables were included in a multivariate model, where the PASI score remained significant explanatory variable (Figure 3A and Supplementary Table 4), although the correlation between PASI and Shannon index was very weak. In this analysis, BSA scores have not been included due to their collinearity with PASI. Bray–Curtis distance-based redundancy analysis (dbRDA) was used to evaluate the effect of the showed that, in all parameters, age (*p* = 0.007), marital status (*p* = 0.003), WHR (*p* = 0.011), BSA (*p* = 0.001), and PASI (*p* = 0.002) were significantly associated with beta-diversity of skin microbiota. However, other environmental factors were not significantly correlated with the skin microbiota (*p* > 0.05, data not shown).

Furthermore, we emphatically analyzed differences in taxonomic composition in relation to psoriasis disease activity scores (PASI and BSA) that significantly affected beta-diversity of the skin microbiota composition. The skin microbiota fractions at all taxonomic levels were compared between moderate-to-severe psoriasis (PASI ≥ 10) and mild psoriasis (PASI < 10; Figure 3B). The volcanic map showed a total of 36 upregulated genera and 1 downregulated gene between the two groups. Nevertheless, the proportions of genera *Klebsiella*, *Phreatobacter*, *Kytococcus*, *Pantoea*, *Blastocatella*, *Luteimonas*, and so on were increased, whereas the genus *Qipengyuania* was decreased in moderate-to-severe psoriasis compared with mild psoriasis. The proportions of various taxa differed between the groups, which indicated that psoriasis skin bacteria are associated with disease activity.

### Biomarkers of skin microbiota can distinguish patients with plaque psoriasis from subtype

**FIGURE 4:**
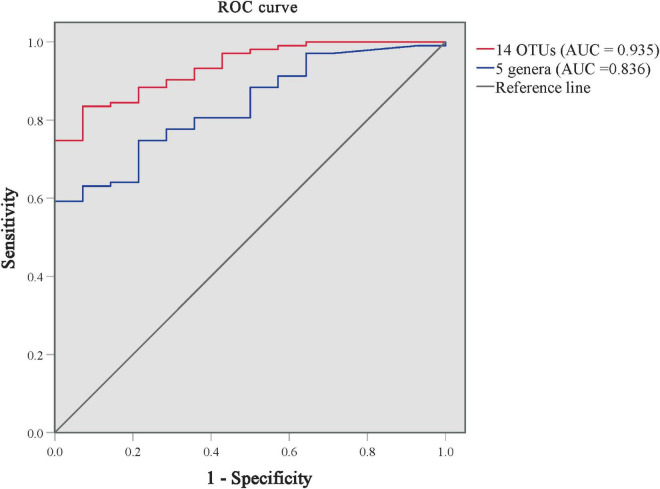
ROC curves for distinguishing GP from PP groups using the models of predominant skin microbiota. The ROC curve of 14 predominant OTUs is in red, and the ROC curve of 5 genera is in blue. AUC, the area under the receiver-operating characteristic curve.

As shown in Supplementary Table 5 and Figure 4, we performed the ROC analysis testing 16 OTUs (at least above genus level) of skin microbiota from the LEfSe results. The predictive abilities of the family *Halomonadaceae* and genus *Halomonas* were low (AUC = 0.335 and 0.333, respectively). The high AUC of 0.935 (95CI%: 0.885–0.985, *p* < 0.001) was obtained with a model including the other 14 OTUs, but an AUC of 0.836 (95CI%: 74.8–92.3%, *p* < 0.001) was already obtained with a model including only five genera (*Staphylococcus*, *Stenotrophomonas*, *Enhydrobacter*, *Brevundimonas*, and *Allorhizobium*–*Neorhizobium*–*Pararhizobium*–*Rhizobium*). The results indicated that the predominant skin microbiota could be considered a potential biomarker for effectively distinguishing patients with PP from GP.

## Discussion

Evidence linking skin and pharyngeal microbiota dysbiosis to chronic inflammation and immune system-mediated diseases has been expanding (Paller et al., 2019; Assarsson et al., 2020; Marazzato et al., 2020; Liang et al., 2021). In this study, we aimed to delineate the community structure of the skin and pharyngeal microbiome from patients with PP (subtype LPP and SPP) and GP through 16S rRNA gene sequencing. Our findings suggest that there were differences in skin microbiota between psoriasis subtypes, but no differences in pharyngeal microbiota. The alpha-diversity in skin microbiota was correlated with psoriasis disease severity. The models consisted of differential skin microbial OTUs and genera could effectively distinguish patients with PP from GP. This study also provided the interindividual comparison of skin and pharyngeal samples, which was necessary to evaluate for bacterial translocation from one body habitat to another. We found that certain genera were shared and positively related between skin and pharyngeal habitat in psoriasis

Similar to other previous studies of psoriasis skin composition (Alekseyenko et al., 2013; Chang et al., 2018), our results showed the three commonest prominent phyla in psoriatic skin: *Proteobacteria*, *Firmicutes*, and *Actinobacteria*. In the psoriatic pharyngeal habitat, the majority of bacteria were identified within the phyla *Firmicutes* and *Bacteroidetes*, in agreement with previous studies (Assarsson et al., 2020).

Later, we evaluated the skin and pharyngeal microbiota alpha-diversity and confirmed significantly lower in patients with SPP than that in patients with LPP, whereas there were no significant differences in alpha-diversity of pharyngeal microbiota among psoriasis subtypes. We also found that the skin microbiota differences of psoriasis subtypes in alpha-diversity resulted from the extent of psoriatic skin lesions (PASI and BSA), consistent with previous research (Alekseyenko et al., 2013). In addition, patients with LPP had higher PASI and BSA scores compared to the SPP and GP groups. Such results suggest that the significant difference of alpha-diversity between SPP and LPP might be caused by the different disease severity.

Beta-diversity analysis showed a similar clustering of skin microbiota samples between patients with LPP and SPP compared to patients with GP. Although PP is subdivided into SPP and LPP according to the size of lesions and thickness of plaques (Griffiths et al., 2007), both LPP and SPP essentially belong to the same psoriasis subtype from their skin microbiota profile. However, pharyngeal microbiota features were not subtype-specific in psoriasis. In addition, LEfSe analyses revealed that the dominant skin microbiota of the PP group included *Staphylococcus*, *Stenotrophomonas*, etc., whereas the GP group included *Halomonas*. Lewis et al. (2019) reported that *S. aureus* colonizes psoriatic lesions in 60% of the patients, and it has a potential capability to initiate psoriasis through upregulating a Th17 response (Chang et al., 2018). *Stenotrophomonas maltophilia* was a potential pathogenic cause of intact skin infection syndromes, especially in immunocompromised patients (Bin Abdulhak et al., 2009). The dysbiosis of skin microbial community could get involved in the pathogenesis of psoriasis (Muszer et al., 2015; Olejniczak-Staruch et al., 2021).

Based on the microbial markers for PP and GP discrimination, the resulting models comprising 16 OTUs and 5 genera performed well (AUC = 0.935 and 0.836, respectively) to distinguish patients with PP from GP. A previous research had demonstrated the microbiome signatures for the diagnosis of psoriasis results with AUC values ranging from 0.75 to 0.89, which covered subjects of both genders, different age groups, ethnicities, and multiple body sites (Statnikov et al., 2013). Future systematic investigations of these OTU markers would be of value in differential diagnosis, diagnosis, and prognosis of psoriasis.

Despite the great variability between the skin and pharyngeal bacterial communities, certain taxa were positively correlated between skin and pharynx habitats in our study. This result could be explained by the hypothesis of bacterial translocation or selection by the immune system (Jin et al., 2022). Many studies of *Streptococcus* detected in both the blood and pharynx of patients with psoriasis provide plausibility to the hypothesis (Zhao et al., 2005; Munz et al., 2010). But recently, Thrastardottir et al. (2022) found a strong site-specific association between pharyngeal cultures and an increased risk of psoriasis, regardless of the pathogen. We guess that *Streptococcus* may act as one of the bacterial triggers, and there are other unidentified pathogenic bacteria transmission from the skin to the pharynx or otherwise, contributing to psoriasis progress.

Some limitations of this study merit our consideration. First, the study is performed at a single medical center in China with a cross-sectional design. Second, the relatively small sample size of patients with GP limited opportunities for random forest analysis, although the overall sample size was enough. Hence, to evaluate the influence of potential confounders in psoriasis, we conducted the dbRDA analysis, which indicated that patient’s age, marital status, WHR, and disease severity were confounders of the associations between skin microbiome composition and psoriasis. Finally, characterization of the skin and pharyngeal microbiota in the present work was performed using 16S rRNA sequencing, and it limits the ability to determine microbial strain purity. Further mechanistic studies should include shotgun metagenomic approaches (Ranjan et al., 2016; Grogan et al., 2019).

In conclusion, this study highlights the different skin microbial community compositions between patients with PP and GP types, but not in pharyngeal microbiota. The skin microbiota may have a certain correlation with the pharyngeal microbiota. Alpha-diversity in skin microbiota was correlated with psoriasis disease severity. The combination of five skin genera (*Staphylococcus*, *Stenotrophomonas*, *Enhydrobacter*, *Brevundimonas*, and *Allorhizobium*–*Neorhizobium*–*Pararhizobium*–*Rhizobium*) may act as a potential marker for distinguishing patients with PP and GP subtypes. Our study provides a new direction for future studies aiming to understand the host-skin microbiota interplay in psoriasis progression.

## Data availability statement

The data presented in this study are deposited in the NCBI BioProject repository, accession number: PRJNA835606.

## Ethics statement

The studies involving human participants were reviewed and approved by the Ethics Committee of the Xiangya Hospital of Central South University. Written informed consent to participate in this study was provided by the participants’ legal guardian/next of kin. Written informed consent was obtained from the individual(s), and minor(s)’ legal guardian/next of kin, for the publication of any potentially identifiable images or data included in this article.

## Author contributions

WZ and YK conceptualized the study. JH and WL designed and acquired the data. JH wrote the manuscript. JC guided the design of study and revised the manuscript. XL, MT, and JY carried out the data analysis. KH, QW, SD, and YL provided help in collecting literature. All authors have read and agreed to the published version of the manuscript.

## Footnotes

1
http://drive5.com/uparse/

2
https://www.ncbi.nlm.nih.gov/sra
