# Supplementary material for: Microbiota differences of skin and pharyngeal microbiota between patients with plaque and guttate psoriasis in China
Source: Front Microbiol. 2022 Aug 10;13:937666. doi: 10.3389/fmicb.2022.937666 (PMC9399812; doi:10.3389/fmicb.2022.937666)
Supplement: Supplementary file 1 [file Data_Sheet_1.docx]

Supplementary Material

# Supplementary Data

## Supplementary Excel 1. Quality Contral Table

## Supplementary Excel 2. OTUs Table

# Supplementary Figures and Tables

## Supplementary Figures


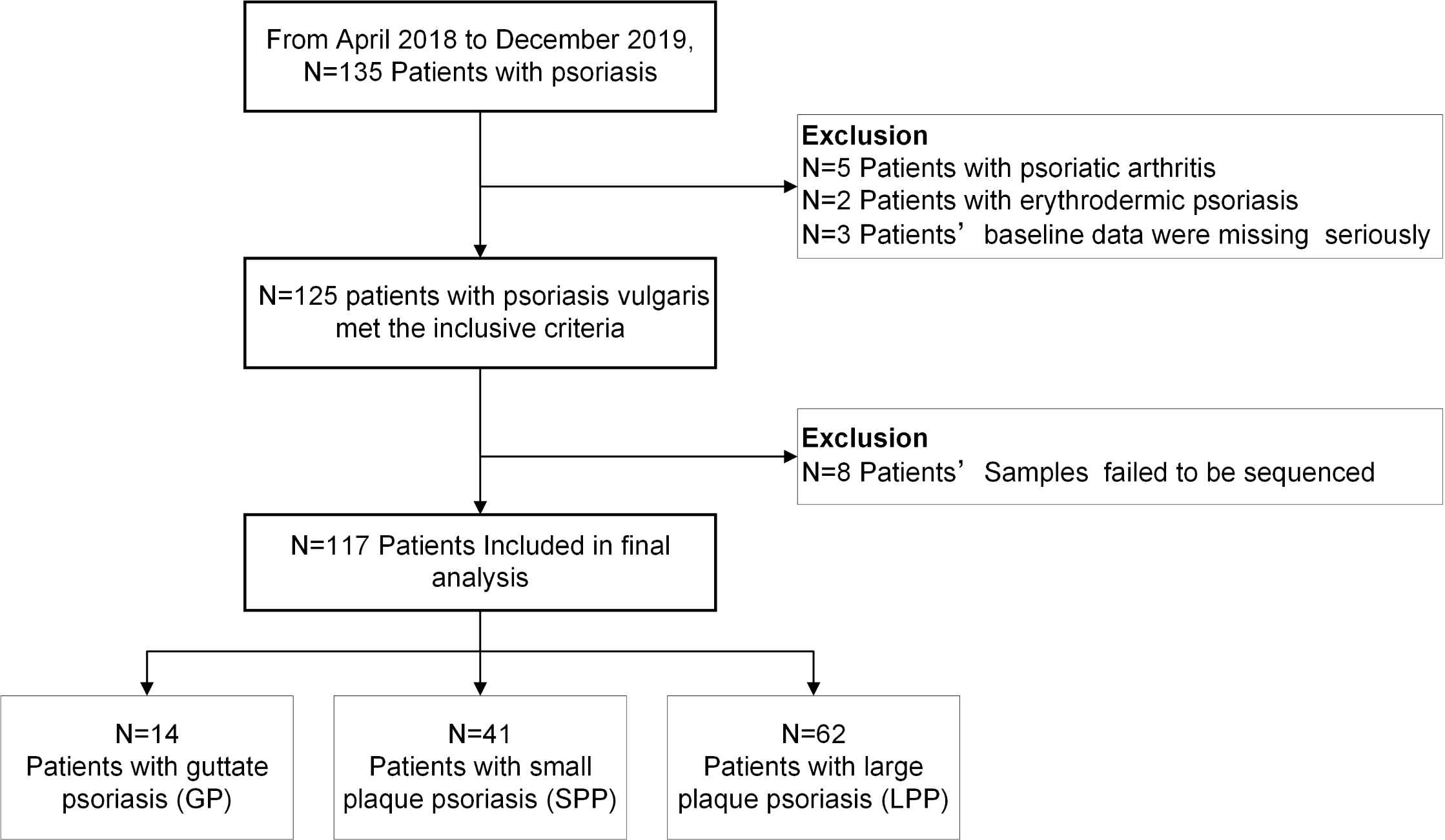
**Supplementary Figure 1.** Flowchart of patient included and excluded studies


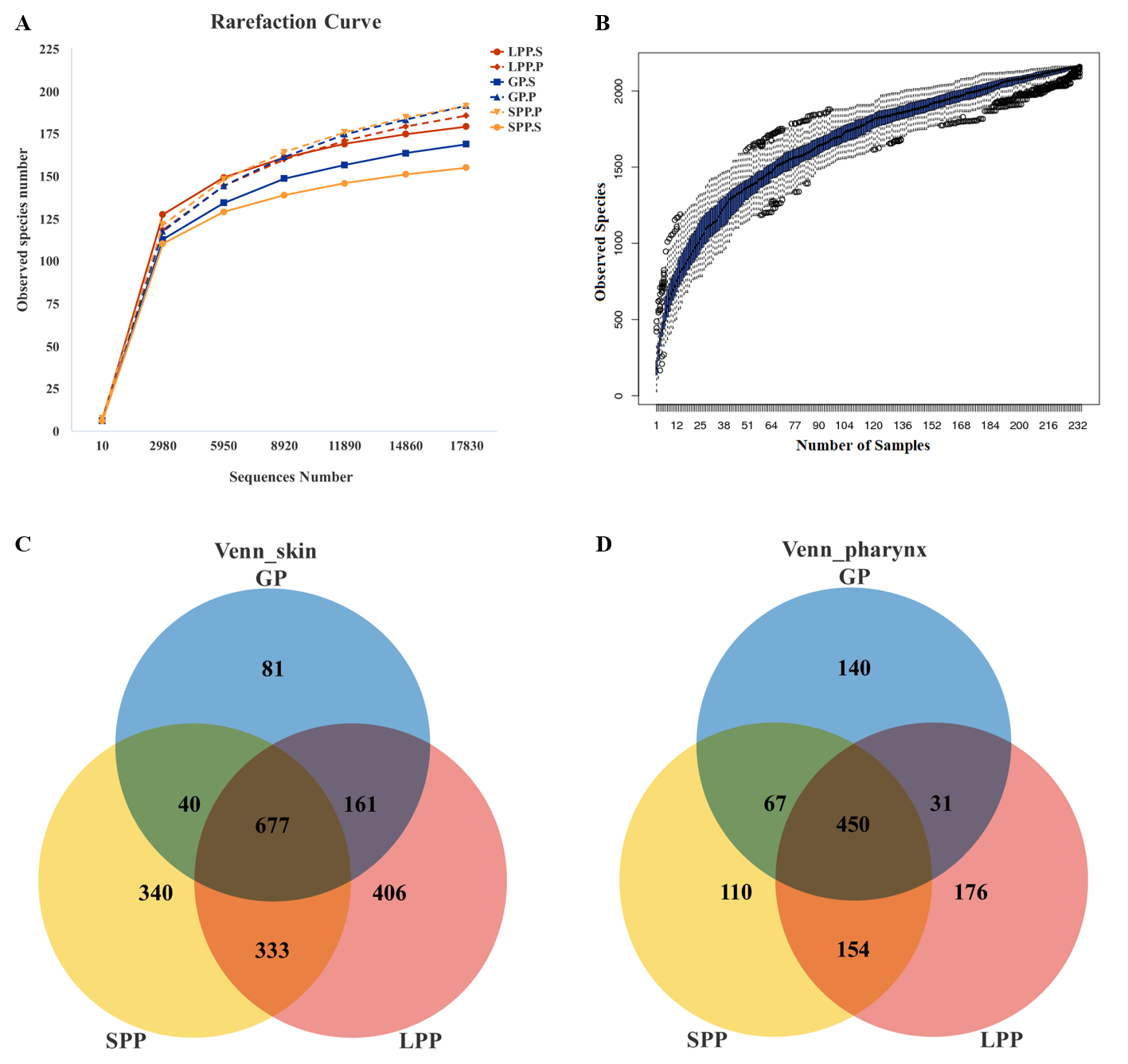


**Supplementary Figure 2.** Estimation of sample depth and Venn diagram of the skin and pharynx microbiota of the GP, SPP, and LPP groups of psoriasis individuals. **(A)** Rarefaction curve of OTUs on each group of samples. Solid lines refer to skin microbial groups and dotted lines refer to pharyngeal microbial groups. **(B)** Species accumulation boxplot. The abscissa represents the sample size and the ordinate represents the number of OTU after sampling. The results reflect the rate of new OTU under continuous sampling. Venn diagram of OUTs clustering from skin samples **(C)** and pharyngeal samples **(D)**. Venn diagrams show the overlapping and unique OTUs between the different groups. Abbreviations: GP, guttate psoriasis; SPP, small plaque psoriasis; LPP, large plaque psoriasis. OUT, operational taxonomic unit.

**
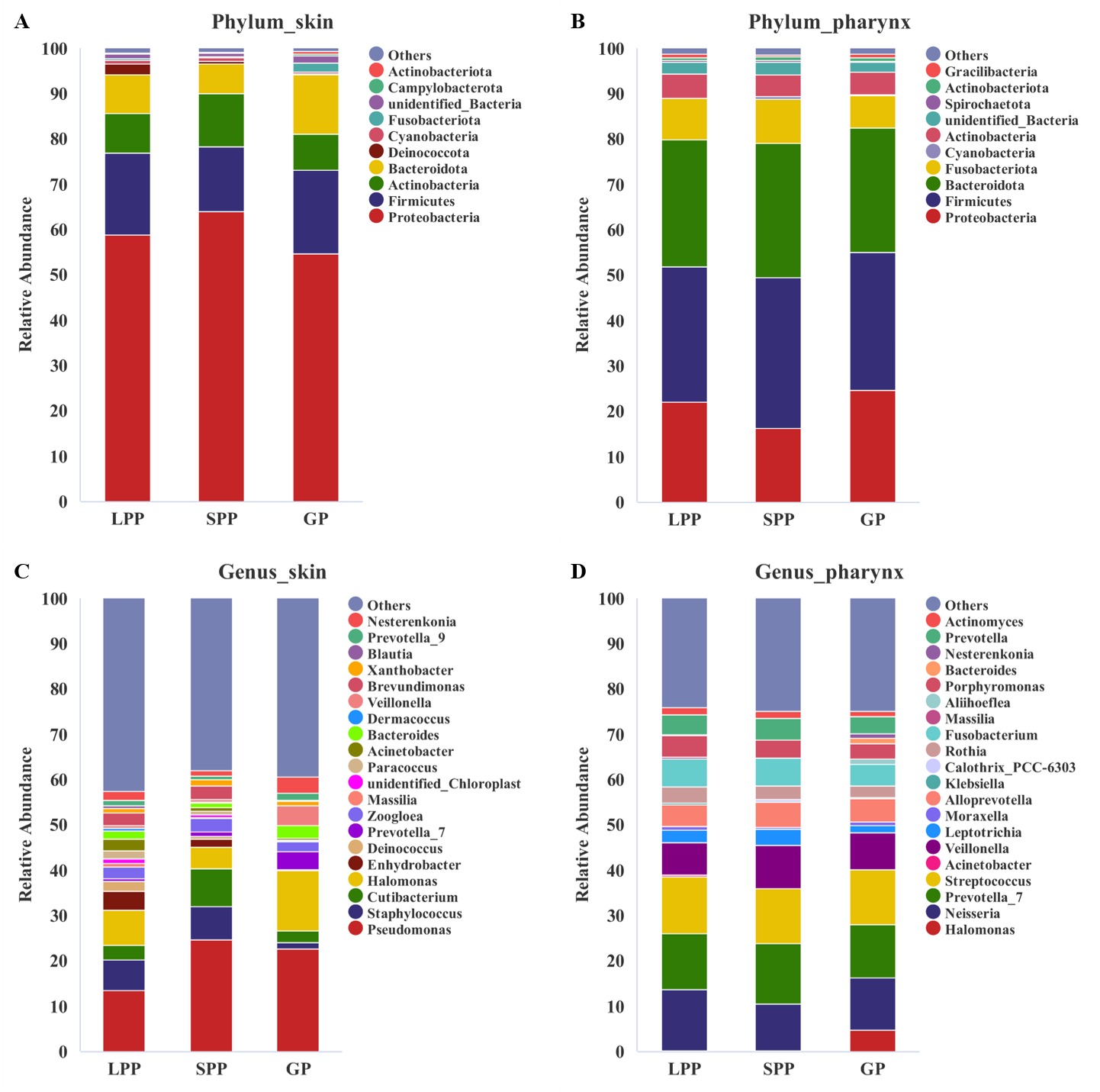
**

**Supplementary Figure 3.** Comparison of the construction of the skin and pharyngeal microbiota among the GP, SPP, and LPP subjects. Relative abundance of top 10 species at phylum level in a cylindrical accumulative graph of skin **(A)** and pharyngeal **(B)** bacteria among three groups. Relative abundance of top 20 species at genus level in the skin **(C)** and pharyngeal **(D)** bacteria among three groups. Others represent the sum of the relative abundance in the other bacteria except for the above top 10 phyla or top 20 genera.

**
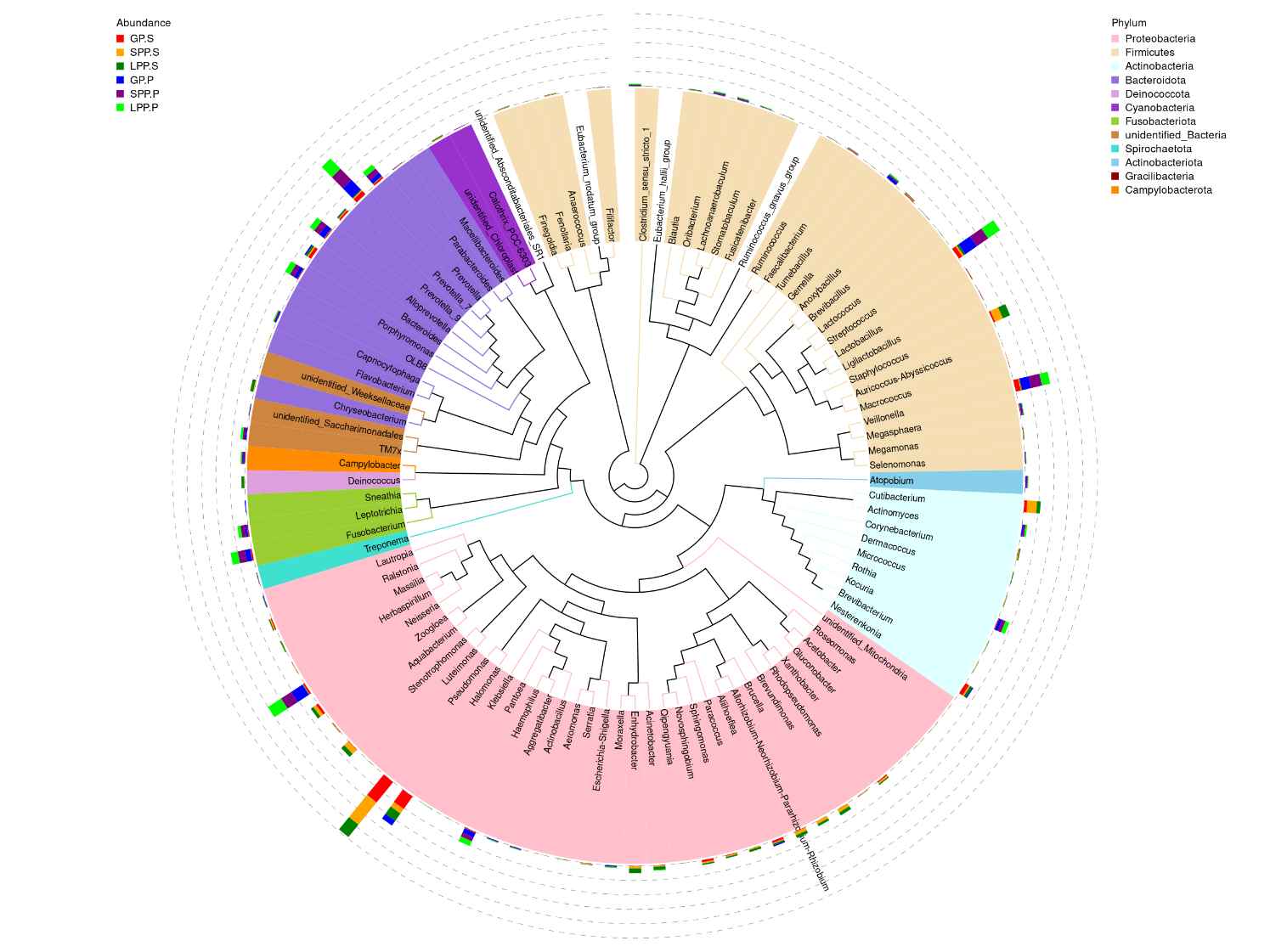
**

**Supplementary Figure 4.** The evolutionary tree representing the top 100 genera. Different colors of the branches indicate different phyla. Relative abundance of each genus in each group is displayed outside the circle with different colors denoting different subtypes.

**
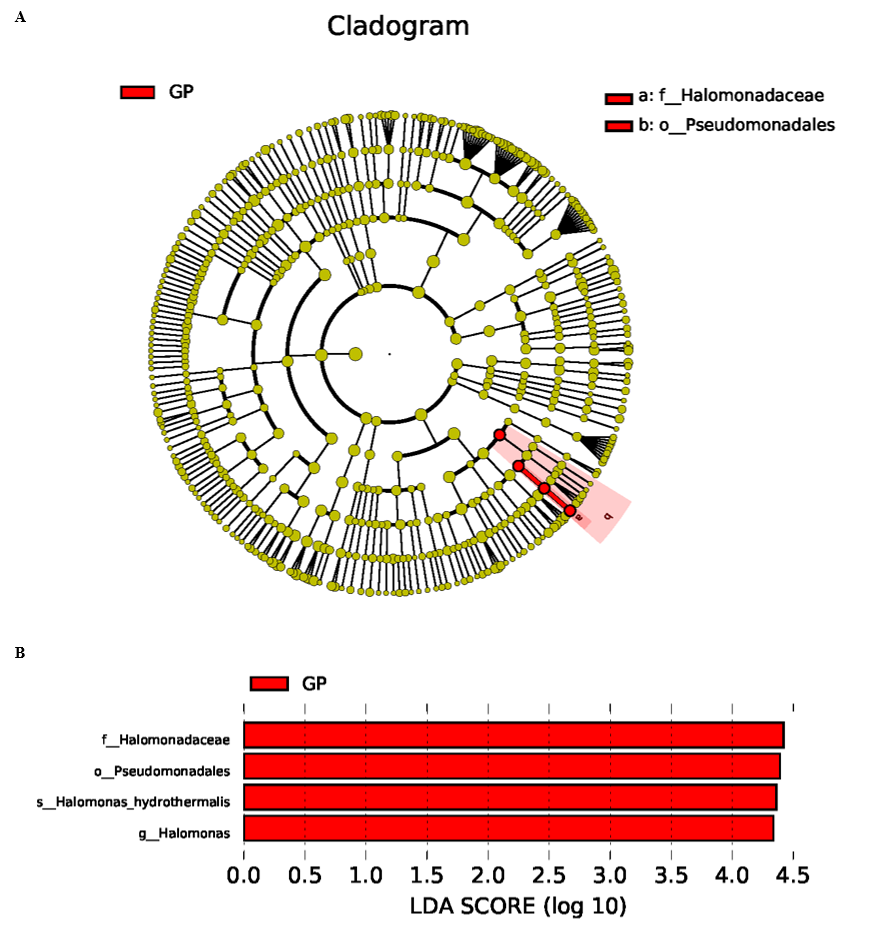
**

**Supplementary Figure 5.** Composition and Linear discriminant analysis of pharyngeal microbiota between PP and GP groups. **(A)** Cladogram generated from the LEfSe analysis indicating the phylogenetic distribution of the microbiota of PP and GP groups from phylum to genus. **(B)** Histogram of LDA scores to identify differentially abundant bacteria between PP and GP (LDA score > 4.0).

## Supplementary Tables

**Supplementary Table 1.** Alpha diversity indices of the skin and pharyngeal microbiota in LPP, SPP and GP patients.

| **Sampling area** | **α-diversity index** | **LPP** | **SPP** | **GP** | ***p-*value** | | |
| --- | --- | --- | --- | --- | --- | --- | --- |
|  |  |  |  |  | **LPP-SPP** | **LPP-GP** | **SPP-GP** |
| **skin** | Chao1 | 197.4±106.0 | 172.4±87.0 | 193.5±114.7 | 0.251 | 0.594 | 0.811 |
|  | Shannon | 4.387±1.137 | 3.747±1.406 | 3.789±1.678 | **0.015^*^** | 0.285 | 0.572 |
|  |  |  |  |  |  |  |  |
| **pharynx** | Chao1 | 214.1±44.0 | 219.9±54.8 | 233.1±59.5 | 0.434 | 0.650 | 0.346 |
|  | Shannon | 4.715±0.466 | 4.781±0.449 | 4.613±0.937 | 0.382 | 0.668 | 0.874 |

Note: PA, primary aldosteronism; SD, standard deviation. * *p* < 0.05.

**Supplementary Table 2.** Predicted KEGG functional pathways differences at level 3 inferred from 16s rRNA gene sequences using PICRUSt2.

| **KEGG pathway categories** | | **GP**  **mean%(SD%)** | **PP**  **mean%(SD%)** | **Fold change*** | ***p*-**  **value** | ***q*-**  **value** |
| --- | --- | --- | --- | --- | --- | --- |
| **Genetic Information Processing** | |  |  |  |  |  |
| Translation | Ribosome biogenesis in eukaryotes | 0.056 (0.007) | 0.05 (0.056) | 0.90 | 0.005 | 0.132 |
| **Human Diseases** | |  |  |  |  |  |
| Metabolic Diseases | Type II diabetes mellitus | 0.043 (0.007) | 0.037 (0.043) | 0.87 | 0.002 | 0.132 |
| **Metabolism** | |  |  |  |  |  |
| Biosynthesis of Other Secondary Metabolites | beta-Lactam resistance | 0.03 (0.006) | 0.04 (0.03) | 1.33 | 0.002 | 0.132 |
| Energy Metabolism | Methane metabolism | 0.996 (0.082) | 0.924 (0.996) | 0.93 | 0.006 | 0.132 |
| Energy Metabolism | Oxidative phosphorylation | 1.236 (0.085) | 1.32 (1.236) | 1.07 | 0.006 | 0.132 |
| Glycan Biosynthesis and Metabolism | Lipopolysaccharide biosynthesis | 0.388 (0.101) | 0.301 (0.388) | 0.78 | 0.005 | 0.132 |
| Lipid Metabolism | Fatty acid biosynthesis | 0.473 (0.031) | 0.499 (0.473) | 1.06 | 0.006 | 0.132 |
| Metabolism of Terpenoids and Polyketides | Carotenoid biosynthesis | 0.017 (0.018) | 0.037 (0.017) | 2.11 | 0.003 | 0.132 |
| Metabolism of Cofactors and Vitamins | Lipoic acid metabolism | 0.049 (0.011) | 0.061 (0.049) | 1.25 | 0.004 | 0.132 |
| Xenobiotics Biodegradation and Metabolism | Naphthalene degradation | 0.216 (0.067) | 0.265 (0.216) | 1.23 | 0.003 | 0.132 |
| Xenobiotics Biodegradation and Metabolism | Ethylbenzene degradation | 0.062 (0.024) | 0.082 (0.062) | 1.31 | 0.004 | 0.132 |

Note: At KEGG level 3, the difference of KEGG pathways in median relative abundance between PP and GP is presented generated using Wilcoxon rank-sum test followed by FDR adjustment with Benjamini–Hochberg method. The threshold FDR (*q*-value) ≤ 0.15 and *p*-value<0.05 were considered statistically significant. *The fold change was calculated as the mean relative abundance ratio between PP and GP groups. Abbreviations: PP, plaque psoriasis; GP, guttate psoriasis; KEGG, Kyoto Encyclopedia of Genes and Genomes; PICRUSt, Phylogenetic Investigation of Communities by Reconstruction of Unobserved States; SD: standard deviation.

**Supplementary Table 3.** Univariate correlations of Psoriasis Patients’ Demographics and Clinical Characteristics with Skin Microbial Shannon index.

| **Variables** | **Correlation Coefficient** | ***p* value** |
| --- | --- | --- |
| **Pearson’s correlation** | r |  |
| Age | 0.169 | 0.072 |
| WHR | 0.139 | 0.138 |
| Age at onset | 0.185 | **0.048^*^** |
| Duration of disease | 0.081 | 0.387 |
| BSA | 0.253 | **0.006^**^** |
| PASI | 0.311 | **<0.001^***^** |
| DLQI | 0.130 | 0.168 |
| **Kendall****’s tau-b correlation** | τ |  |
| Gender | 0.037 | 0.631 |
| Marital status | 0.173 | **0.024^*^** |
| Educational level | -0.034 | 0.643 |
| Smoking | 0.065 | 0.379 |
| Drinking | -0.106 | 0.148 |
| BMI categories | 0.148 | **0.048^*^** |
| Family history of psoriasis | 0.074 | 0.337 |

Note: Pearson linear correlation coefficient (r) and Kendall’s tau-b rank correlation coefficient (τ) were used for correlation analyses, respectively. Abbreviations: BMI, body mass index; WHR, waist-to-hip ratio; PASI, psoriasis area and severity index; BSA, body surface area; DLQI, dermatology life quality index. Values in bold are significant values at p-value <0.05 in multivariable analysis. * *p* < 0.05; ** *p* < 0.01; *** *p* < 0.001.

**Supplementary Table 4.** Multivariate linear regression analysis of Psoriasis Patients’ Demographics and Clinical Characteristics with Skin Microbial Shannon index.

| **Variables** | **Coefficient (SE)** | ***p* value** |
| --- | --- | --- |
| Married | 0.437 (0.330) | 0.189 |
| Underweight | -0.471 (0.473) | 0.321 |
| Overweight | 0.150 (0.282) | 0.595 |
| PASI | 0.038 (0.012) | **0.002^*^** |
| Age at onset | 0.004 (0.011) | 0.718 |
| F | 3.618 | |
| *p* value | **0.005^**^** | |
| R^2^ | 0.142 | |

Note: *P* values were <0.05 for all variables from the univariate correlations analyses entered in the multivariable analysis. *P* values for BSA were <0.05 but it wasn’t included in the multivariable analysis because of collinearity of BSA and PASI (VIF, 5.087 and 5.146). Values in bold are significant values at p-value <0.05 in multivariable analysis. SE, Standard Error. PASI: Psoriasis Area Severity Index. * *p* < 0.05; ** *p* < 0.01; *** *p* < 0.001.

**Supplementary Table 5. AUC of different biomarkers for distinguish PP from GP.**

|  | **ACU (95%CI)** | ***p-*value** | **Sensitivity** | **Specificity** | **Youden Index** |
| --- | --- | --- | --- | --- | --- |
| o_Caulobacterales | 0.818 (0.716-0.920) | 0.000 | 0.806 | 0.786 | 0.592 |
| f_Moraxellaceae | 0.817 (0.725-0.909) | 0.000 | 0.650 | 0.929 | 0.579 |
| f_Caulobacteraceae | 0.816 (0.714-0.918) | 0.000 | 0.806 | 0.786 | 0.592 |
| g_Brevundimonas | 0.785 (0.683-0.887) | 0.001 | 0.728 | 0.857 | 0.585 |
| g_Staphylococcus | 0.771 (0.630-0.913) | 0.001 | 0.922 | 0.571 | 0.494 |
| f_Staphylococcaceae | 0.761 (0.610-0.912) | 0.002 | 0.922 | 0.571 | 0.494 |
| f_Xanthomonadaceae | 0.739 (0.616-0.862) | 0.004 | 0.845 | 0.571 | 0.416 |
| c_Alphaproteobacteria | 0.731 (0.578-0.884) | 0.005 | 0.854 | 0.571 | 0.426 |
| g_Allorhizobium-Neorhizobium-Pararhizobium-Rhizobium | 0.728 (0.607-0.849) | 0.006 | 0.466 | 0.929 | 0.395 |
| g_Stenotrophomonas | 0.725 (0.596-0.855) | 0.006 | 0.845 | 0.571 | 0.416 |
| o_Staphylococcales | 0.725 (0.581-0.868) | 0.007 | 0.641 | 0.786 | 0.426 |
| g_Enhydrobacter | 0.713 (0.592-0.835) | 0.010 | 0.398 | 1.000 | 0.398 |
| o_Rhizobiales | 0.681 (0.533-0.828) | 0.029 | 0.932 | 0.357 | 0.289 |
| f_Rhizobiaceae | 0.676 (0.542-0.810) | 0.033 | 0.369 | 0.929 | 0.298 |
| f_Halomonadaceae | 0.335 (0.169-0.501) | 0.046 | 0.010 | 1.000 | 0.010 |
| g_Halomonas | 0.333 (0.167-0.498) | 0.043 | 0.010 | 1.000 | 0.010 |
